# S-15 in combination of Akt inhibitor promotes the expansion of CD45RA^−^CCR7^+^ tumor infiltrating lymphocytes with high cytotoxic potential and downregulating PD-1^+^Tim-3^+^ cells as well as regulatory T cells

**DOI:** 10.1186/s12935-019-1043-3

**Published:** 2019-12-03

**Authors:** Benling Xu, Long Yuan, Guangyu Chen, Tiepeng Li, Jinxue Zhou, Chengjuan Zhang, Peng Qin, Musleh M. Muthana, Shengdian Wang, Xuexiang Du, Quanli Gao

**Affiliations:** 1 Department of Immunotherapy, The Affiliated Cancer Hospital of Zhengzhou University, Zhengzhou, Henan People’s Republic of China; 2 Department of General Surgery, The Affiliated Cancer Hospital of Zhengzhou University, Zhengzhou, Henan People’s Republic of China; 3 Department of Hepatobiliary and Pancreatic Surgery, The Affiliated Cancer Hospital of Zhengzhou University, Zhengzhou, Henan People’s Republic of China; 4 Division of Immunotherapy, Institute of Human Virology, University of Maryland, Baltimore, MD 21201 USA; 5 CAS Key Laboratory of Infection and Immunity, Institute of Biophysics, Chinese Academy of Sciences, Beijing, China

**Keywords:** Hepatocellular cancer, Tumor infiltrating T lymphocytes, Akt inhibitor, Central memory T cells, Regulatory T-cells, Programmed death 1

## Abstract

**Background:**

Autologous tumor-infiltrating lymphocytes (Tils) immunotherapy is a promising treatment in patients with advanced hepatocellular cancer. Although Tils treatment has shown great promise, their persistence and the efficacy after adoptive-transfer are insufficient and remain a challenge. Studies have demonstrated that IL-15 and Akt inhibitor can regulate T cell differentiation and memory. Here, we constructed S-15 (Super human IL-15), a fusion protein consisting of human IL-15, the sushi domain of the IL-15 receptor α chain and human IgG-Fc. Herein we compared the effects of S-15 with IL-2 or in combination with Akti on the expansion and activation of Tils.

**Methods:**

Hepatocellular cancer tissues were obtained from 6 patients, Tils were expanded using IL-2, IL-2/S-15, IL-2/Akti or in combination IL-2/S-15/Akti. At day 10, anti-CD3 antibody was added to the culture media and expanded to day 25. The composition, exhaustion and T-cell differentiation markers (CD45RA/CCR7) were analyzed by flow cytometry.

**Results:**

We found that IL-2/S-15/Akti expanded Tils and showed the highest percentage of central memory CD45RA^−^CCR7^+^ phenotype prior to anti-CD3 antibody activation and after anti-CD3 antibody activation. T cells cultured with IL-2/S-15/Akti exhibited a mixture of CD4^+^, CD8^+^, and CD3^+^CD4^−^CD8^−^ T cells; S-15 in combination with Akt inhibitor downregulated the expression of PD-1^+^Tim-3^+^ on Tils and decreased the Tregs in Tils. Additionally, the Tils expanded in the presence of the Akt inhibitor and S-15 showed enhanced antitumor activity as indicated by the increase in IFN-γ producing tumor infiltrating CD8^+^ T cells and without comprising the Tils expansion.

**Conclusion:**

Our study elucidates that IL-2/S-15/Akti expanded Tils and represent a viable source for the cellular therapy for patients with hepatocellular cancer.

## Background

Cellular therapy using autologous tumor-infiltrating lymphocytes (Tils) is a promising strategy to improve the survival of patients with primary hepatocellular carcinoma (HCC). Reports have shown that up to 80% of HCC patients who received transferred autologous Tils have shown positive clinical response [1]. While the use of Tils have shown improved clinical response and survival of HCC patients, insufficient persistence and effector function of Tils in vivo remain a challenge for adoptive T cell therapy. Recent studies have shown that the transfer of Tils with central memory features can improve the efficacy and the curative potential of Tils for advanced cancer [2, 3]. Akt plays an important role in regulating T cells differentiation and memory formation. Inhibition of Akt signaling during ex vivo priming and expansion enhances expansion of potent tumor-specific lymphocytes with memory cell characteristics [4–7].

Studies indicate that cytokines have a distinct effect on different T-cells subsets [8–12]. Although IL-2 and IL-15 share the cognate receptors IL-2Rβ and IL-2Rγ, IL-2 is widely used for T cell growth and is required for in vitro growth of CD4^+^ T cells. IL-15 can improve CD8^+^ T cells function in vivo and plays a pivotal role in the control of memory T cells by inducing anti-apoptotic signals and promotes proliferation dependent on PI3K/Akt, MAPK, and ERK pathways [13]. Recent reports have shown that the administration of IL-15 with its soluble receptor IL-15Rα complex enhanced the bioavailability of IL-15 by about 50-fold and showed elevated tumor-eliminating effects in vivo [14, 15]. Liu et al. have shown that IL-2/IL-15/IL-21 expanded Tils represent a viable source for the cellular therapy of patients with gliomas [16]. However, the optimal protocols for expansion of Tils remain to be determined.

We therefore hypothesized that S-15 which is composed of human IL-15 and the sushi domain of the IL-15 receptor α chain as a hIgG-Fc fusion protein in combination with Akt inhibitor may prevent terminal differentiation and enhance the antitumor activity of Tils during expansion. To evaluate this hypothesis, we constructed and purified S-15, then expanded Tils in the presence of an Akt inhibitor and S-15.

We found that IL-2/S-15/Akti expanded Tils showed the highest percentage of central memory CD45RA^−^CCR7^+^ phenotype either before anti-CD3 activation or after activation, S-15 in combination with Akt inhibitor downregulated the expression of PD-1^+^Tim-3^+^ and decreased the Tregs in Tils. Additionally, combination treatment also enhances the antitumor activity of Tils by increasing the IFN-γ producing tumor infiltrating CD8^+^ T cells without affecting expansion.

The use of S-15 and Akti during the ex vivo expansion of Tils can improve their quality and efficacy in cell-based immunotherapy for HCC.

## Materials and methods

### Patients

HCC patients were diagnosed according to pre-operative staging and laparotomy findings between 2016 and 2017 at the Department of Hepatobiliary and Pancreatic Surgery, the Affiliated Cancer Hospital of Zhengzhou University, China. 6 primary HCC patients without previous treatments were qualified and enrolled in this study.

### Vector construction, recombinant protein preparation

S-15 was constructed as described previously [14]. Briefly, the cDNA encoding human IL-15 Ra-sushi domain (amino acids 25-89) and human IL-15 mature sequence were linked by a 20-amino acid linker were sub-cloned into the ptt3 plasmid. Construction of the human S-15 is shown in Additional file 1: Fig. S1a. The protein was prepared by transient transfection of 293T cells and purified by protein G columns.

### Isolation of Tils and the in vitro culture

The fresh human tumor samples from patients with HCC were cut into small pieces (3–5 mm^3^) and were digested with collagenase (1 μg/ml, Sigma-Aldrich, St. Louis, MO, USA), DNase (25 μg/ml; Sigma-Aldrich), and 2% fetal bovine serum at 37 °C for 1–1.5 h. The tissue homogenates were filtered using a 70-μm cell strainer (Falcon; BD Biosciences) and subjected to the density centrifugation. The leukocyte’s viability was evaluated by Trypan blue exclusion. Tils were cultured at a concentration of 1 × 10^6^ cells/ml with 7000 IU/ml IL-2 in the condition of 10 ng/ml S-15, or 1 μM Akti (Akt1/2 kinase inhibitor, Sigma A6730), or in combination of S-15 and Akti for 10 days, the Tils were then stimulated with precoated anti-CD3 antibody (1.5 μg/ml) and a reduced dose of 1000 IU/ml IL-2 at day 10, fresh S-15 and Akti was added every 72 h for 15 days. After anti-CD3 antibody stimulated, Tils were counted and cultured at a concentration of 2 × 10^5^ cells/ml in a 75 cm^2^ Flask. Then, Tils were transferred to another 75 cm^2^ Flask and continued to culture at a concentration of 2 × 10^5^ cells/ml when the Tils reached to 1–2×10^6^ cells/ml. We repeated the procedure until day 25 to count the total number of TILs. Subsets of Tils were detected by multiple-color fluorescence. Remaining tumor cells were cryopreserved for subsequent analysis.

### Phenotypic analysis

Specific antibodies against CD3-PE-Cy7 (Biolegend) or anti-CD3-FITC (Biolegend), anti-CD25-PE (BD Pharmingen™), anti-CD127-APC (Biolegend), anti-CD4-FITC (Biolegend), anti-CD8-APC (Biolegend), anti-CD45RA-FITC (Biolegend), anti-CCR7-PE-Cy7 (Biolegend), anti-CD4-PE (BD Biosciences), anti-PD-1-FITC (Biolegend), anti-CD4-PerCP-Cy5.5 (BD Biosciences), anti-Tim-3-PE (BD Pharmingen™), anti-FoxP3-PE (Biolegend), and anti-IFN-γ-APC (eBioscience) were used. 7AAD viability staining (Biolegend) was used to assess the viability of the cells. Tils were examined at day 0, 5, 10, 15, 20 and 25 by flow cytometry. Briefly, 1 × 10^6^ T cells was mixed with 5 μl of each antibody and was incubated on ice for 20 min in the dark. After incubation the samples were washed with FACS buffer (5% BSA in PBS, 0.09% sodium azide). The pellets were suspended in 300 μl of FACS buffer and acquired on a BD FACS Conto II flow cytometer, and then the data was analyzed with FlowJo software (TreeStar Inc). The data is displayed as background-corrected values with control sample or by fluorescence minus one (FMO).

### Intracellular IFN-γ staining assay

For IFN-γ production assays, co-culture of Tils with autologous tumor was performed and the generation of IFN-γ producing cells in Tils were analyzed by flow cytometry. Tils co-cultured with autologous tumor for 6 h, and subsequently analyzed for IFN-γ [17]. Brefeldin A (Sigma-Aldrich) was added to the culture medium (10 μg/ml) after 2 h of autologous tumor added. The cells were then stained with anti-CD3-PE-Cy7 and anti-CD8-PerCp-Cy5.5 antibodies, fixed, permeabilized, and then labeled with anti-human IFN-γ-APC (eBioscience). Ten thousand events were collected during flow cytometric analysis.

### Data analyses and statistics

Statistical analysis was performed using GraphPad Prism 5.0 (GraphPad Software, United States). Repeated measures ANOVA was used to statistical significance of differences between groups with a Tukey post-test, if data followed Gaussian distribution. If the data did not pass normality test, a Friedman test was used, followed by a Dunn’s post-test. P value < 0.05 was considered statistically significant, as indicated with asterisks (*P < 0.05, **P < 0.01, ***P < 0.001).

## Results

### Combination of S-15 and Akt inhibition does not compromise Tils expansion

Our previous studies have showed that murine hyper-interleukin15 (hyper-IL-15) can suppress metastatic and autochthonous liver cancer by promoting tumor-specific CD8^+^ T cell responses [14]. Here,we constructed S-15 (ptt3-IL-15Rα-sushi-linker-IL-15-hIgG-Fc) which is composed of human IL-15 and the sushi domain of the IL-15 receptor α chain, the protein was prepared by transient transfection of 293T cells and purified by protein G columns (Additional file 1: Fig. S1b). Then we compared the effects of IL-2, S-15 and Akti on the expansion and activation of Tils.

**Fig. 1:**
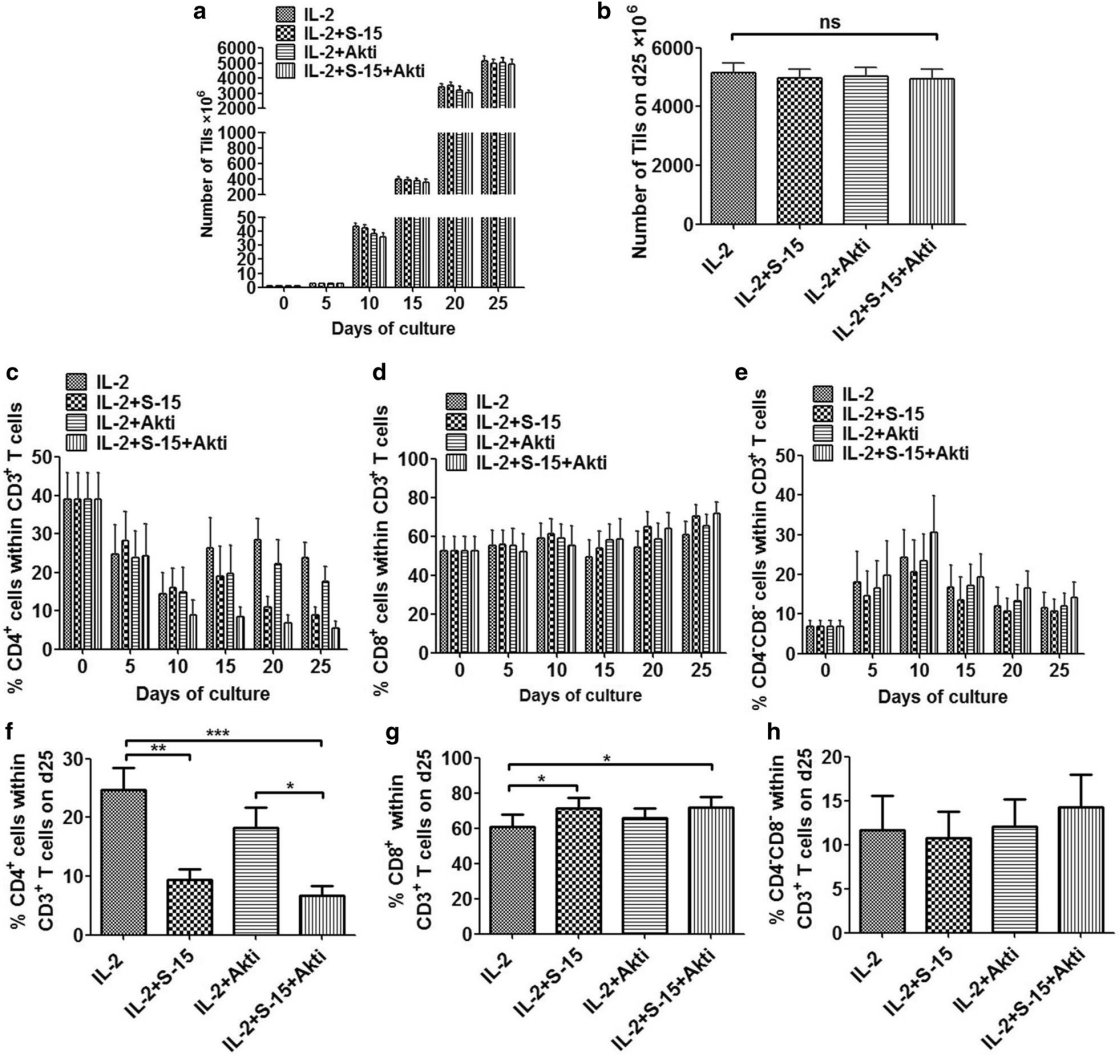
Efficiency of Tils expansion via IL-2, IL-2/S-15, IL-2/Akti and IL-2/S-15/Akti. **a** Growth profiles of Tils from HCC patients during the 25-day initial culture period. Data represent the mean ± SEM of six independent experiments. **b** The total number of Tils in the IL-2, IL-2/S-15, IL-2/Akti and IL-2/S-15/Akti-expanded cultures is depicted for 6 HCC patients. Growth profiles of CD8^+^ T cells (**c**), CD4^+^ T cells (**d**), CD8^−^ CD4^−^ T cells (**e**) within the CD3^+^ population during the 25-day initial culture period. Percentages of CD8^+^ T cells, CD4^+^ T cells, CD8^−^ CD4^−^ T cells within the CD3^+^ population at day 25 were shown respectively in **f**, **g** and **h**. Statistical significance was analyzed by repeated measures ANOVA. *P < 0.05, **P < 0.01, ***P < 0.001

Tils from 6 patients with HCC were isolated and expanded ex vivo in the condition of IL-2, IL-2/S-15, IL-2/Akti or a combination of IL-2/S-15/Akti. Tils were examined at day 0, 5, 10, 15, 20 and 25 by flow cytometry. Tils were all successfully expanded to a minimum of 3.7 × 10^9^ after anti-CD3 antibody stimulation at day 25. There was no significant difference in the number of the expanded Tils among the groups neither before anti-CD3 antibody stimulation nor after anti-CD3 antibody stimulation (Fig. 1a, b).

During the Tils culture, the percentages of CD4^+^ cells were decreased gradually (Fig. 1c), while the percentages of CD8^+^ cells within CD3^+^ T cells were increased gradually (Fig. 1d). The percentages of CD8^−^CD4^−^ cells within CD3^+^ T cells were sharply elevated during the early stage of Tils culture, but gradually decreased towards the initial level (Fig. 1e). After 25 days, the majority of Tils were positive for CD3^+^ T cells. The percentage of CD4^+^ cells among the CD3^+^ T cells on day 25 in group IL-2 was significant higher than that in group IL-2/S-15 (P < 0.01), and group IL-2/S-15/Akti (P < 0.001, Fig. 1f). The percentage of CD8^+^ cells among the CD3^+^ T cells on day 25 in group IL-2/S-15 and group IL-2/S-15/Akti were both significant higher than that in group IL-2 (P < 0.05, Fig. 1g). There was no significant difference in the percentage of CD4^−^CD8^−^ cells among the CD3^+^ T cells on day 25 in the various groups (Fig. 1h).

### Combination of S-15 and Akt inhibition promote the expression of CD45RA^−^CCR7^+^ in human Tils

**Fig. 2:**
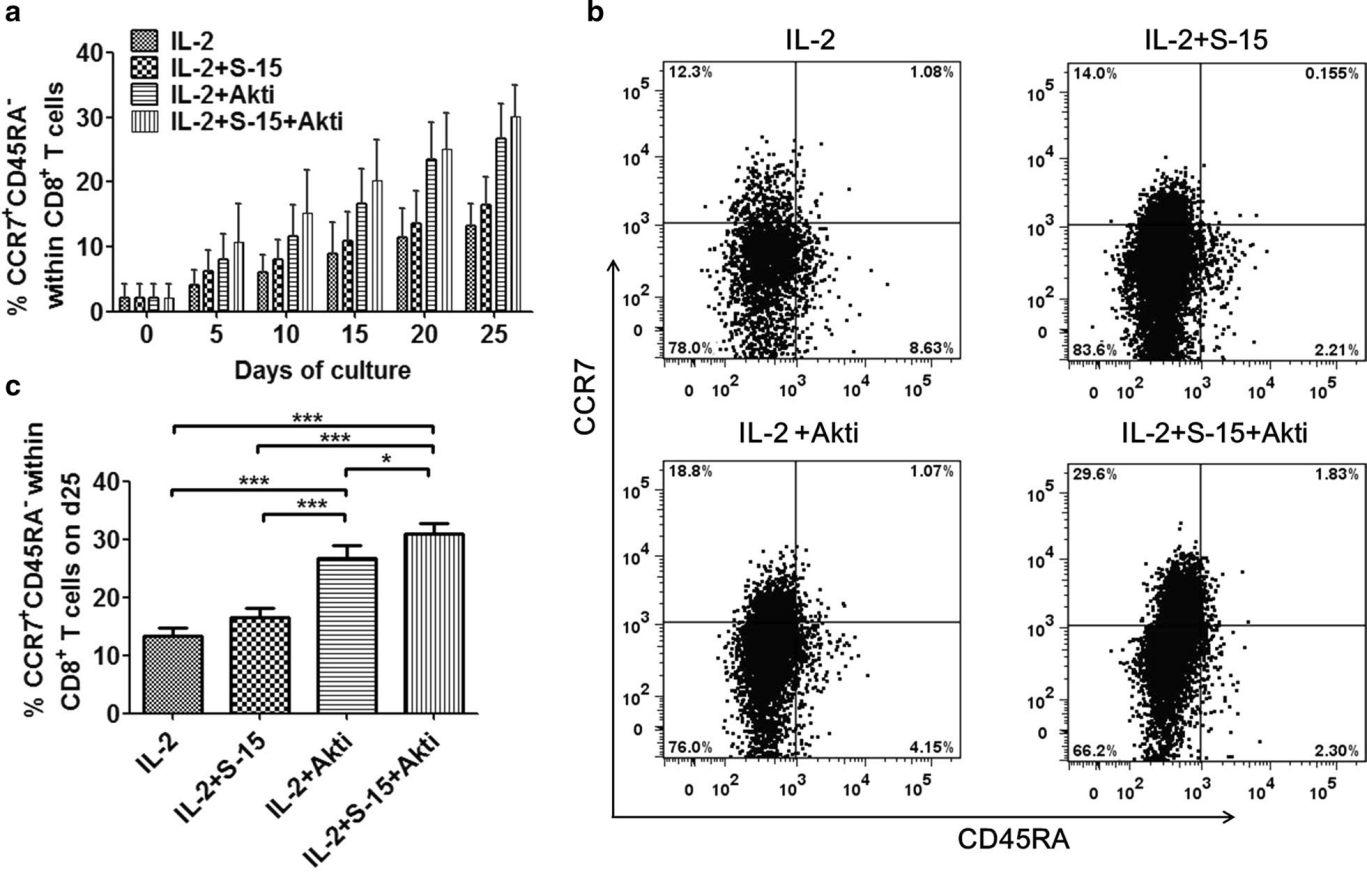
Frequencies of CD45RA^−^CCR7^+^ on CD8^+^ T cells in IL-2, IL-2/S-15, IL-2/Akti and IL-2/S-15/Akti-expanded Tils. **a** The dynamic percentages of Tcm within CD8^+^ T cells during the 25-day initial culture period were shown. Data represent the mean ± SEM of six independent experiments. **b** Representative dot plots with percentages of CD45RA^−^CCR7^+^ among the CD8^+^ T cell population in different groups at day 25 are shown. **c** Summary data about the percentages of CD45RA^−^CCR7^+^ within the CD8^+^ T cell population at day 25 which is from panel **a** are presented. Statistical significance was analyzed by repeated measures ANOVA

Studies report that transfer of Tils with central memory traits can enhance antitumor immunity and have curative potential in adoptive therapy for cancer [18]. IL-15 and Akti both can upregulate the expression of central memory T cells in human Tils. Next, we analyzed the state of T-cell differentiation according to the expression of CCR7 and CD45RA in the various groups. We found that the percentage of Tcm (CD45RA^−^CCR7^+^) among Tils in these four groups all increased gradually during the culture time. The percentage of Tcm within CD8^+^ T cells in group IL-2/S-15/Akti at d25 was significantly higher than that in IL-2 group (P < 0.001), IL-2/S-15 group (P < 0.001) and IL-2/Akti group (P < 0.05, Fig. 2). The percentage of Tcm within CD4^+^ T cells in group IL-2/S-15/Akti at d25 was also significantly higher than that in other three groups, the data is summarized in Additional file 2: Fig. S2. The percentages of Tcm within CD8^+^ T cells in IL-2/Akti group had a similar variation curve to IL-2/S-15/Akti group, it was significantly higher than that in IL-2 group (P < 0.001) and IL-2/S-15 group (P < 0.001) at day 25. The percentages of Tcm among CD8^+^ T cells in IL-2/S-15 group was higher than that in group IL-2 at day 25, but it still lower than that in IL-2/Akti group. However, there was no difference between IL-2 group and IL-2/S-15 group before and after anti-CD3 antibody activation. Thus, supplement with Akt inhibitor enables expansion of Tils expressing elevated levels of Tcm without affecting their expansion.

### Combination of S-15 and Akt inhibition downregulated the expression of PD-1 and Tim-3 on Tils

**Fig. 3:**
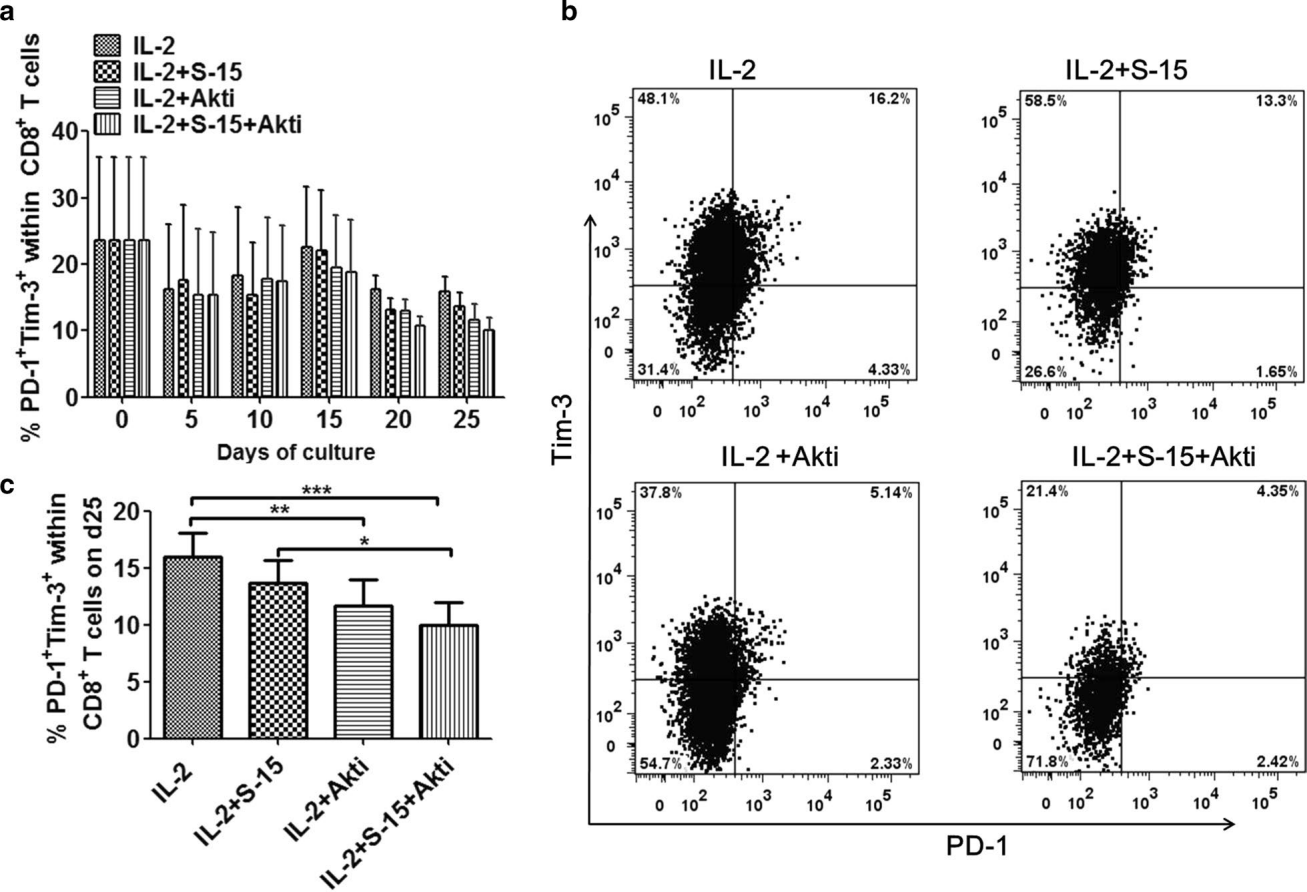
Expression of PD-1 and Tim-3 on CD8^+^ T cells in IL-2, IL-2/S-15, IL-2/Akti and IL-2/S-15/Akti-expanded Tils. **a** The dynamic percentages of PD-1^+^Tim-3^+^ within CD8^+^T cells during the 25-day initial culture period were shown. Data represent the mean ± SEM of six independent experiments. **b** Representative dot plots with percentages of PD-1^+^Tim-3^+^ within CD8^+^T cells in different groups at day 25 are shown. **c** Summary data about the percentages of PD-1^+^Tim-3^+^ within CD8^+^T cells at day 25 which is from panel **a** are presented. Statistical significance was analyzed by repeated measures ANOVA

Programmed death-1 (PD-1) is a key immune-checkpoint receptor expressed by activated T cells mediating immunosuppression, PD-1^+^Tim-3^+^ is a marker of T cells exhaustion [17–19]. In this study, we investigated the expression of inhibitory molecules PD-1 and Tim-3 on Tils during 25 days of incubation with IL-2, IL-2/S-15, IL-2/Akti or in a combination of IL-2/S-15/Akti. The co-expression of PD-1 and Tim-3 on CD4^+^ and CD8^+^ T cells were higher from freshly isolated Tils, then it was decreased sharply at the early stage of ex vivo culture, but it increased after anti-CD3 antibody stimulated, then it decreased gradually to the bottom at day 25 (Fig. 3).

The percentage of PD-1^+^Tim-3^+^ among CD8^+^ T cells in IL-2/S-15/Akti group was significantly lower than that in IL-2 group (P < 0.001) and IL-2/S-15 group (P < 0.05) at day 25. The results for CD8^+^ T cells are showed in Fig. 3, for CD4^+^ T cells, the results are showed in Additional file 3: Fig. S3. The percentage of PD-1^+^Tim-3^+^ among CD8^+^ T cells in group IL-2 was significantly higher than that in IL-2/Akti group (P < 0.01), but there was no significant difference between IL-2/Akti group and IL-2/S-15 group, as well as that between group IL-2 and group IL-2/S-15.

FCM analysis showed that the expression of PD-1 on CD4^+^ and CD8^+^ T cells displayed a similar variation curve to PD-1^+^Tim-3^+^. The percentage of PD-1^+^ at d25 in IL-2/S-15/Akti group was significantly lower than that in IL-2 group (P < 0.001 for CD4^+^ T cells, Additional file 4: Fig. S4b; P < 0.001 for CD8^+^ T cells, Additional file 4: Fig. S4d), and IL-2/S-15 group (P < 0.01 for CD4^+^ T cells; P < 0.05 for CD8^+^ T cells). The percentage of PD-1^+^ within CD4^+^ T cells at d25 in IL-2/S-15/Akti group was significantly lower than that in IL-2/Akti group (P < 0.05), but there was no significant difference about the percentage of PD-1^+^ within CD8^+^ T cells at d25 between IL-2/Akti group and IL-2/S-15/Akti group. The percentage of PD-1^+^ among CD8^+^ T cells in IL-2 group was significantly higher than that in IL-2/Akti group (P < 0.01), but there was no significant difference between IL-2/Akti group and IL-2/S-15 group. There was no significant difference about the percentage of PD-1^+^ among CD4^+^ T cells between IL-2 group and IL-2/S-15 group.

The expression of Tim-3 remained at a high level over the entire incubation period. The percentage of Tim-3^+^ on CD8^+^ T cells in the IL-2/S-15/Akti group was significantly lower than that in IL-2 group (P < 0.01), and IL-2/S-15 group (P < 0.01) at d25 (Additional file 5: Fig. S5d). No significant difference in the percentage of Tim-3^+^ within CD4^+^ T cells was observed among groups.

These results showed that Akt inhibitor and S-15 had synergistic effect on decreasing the expression of PD-1 and Tim-3 in Tils, indicating Akti and S-15 can prevent Tils cell differentiation and exhaustion.

### Combination of S-15 and Akt inhibition decreases the Treg cells in Tils

**Fig. 4:**
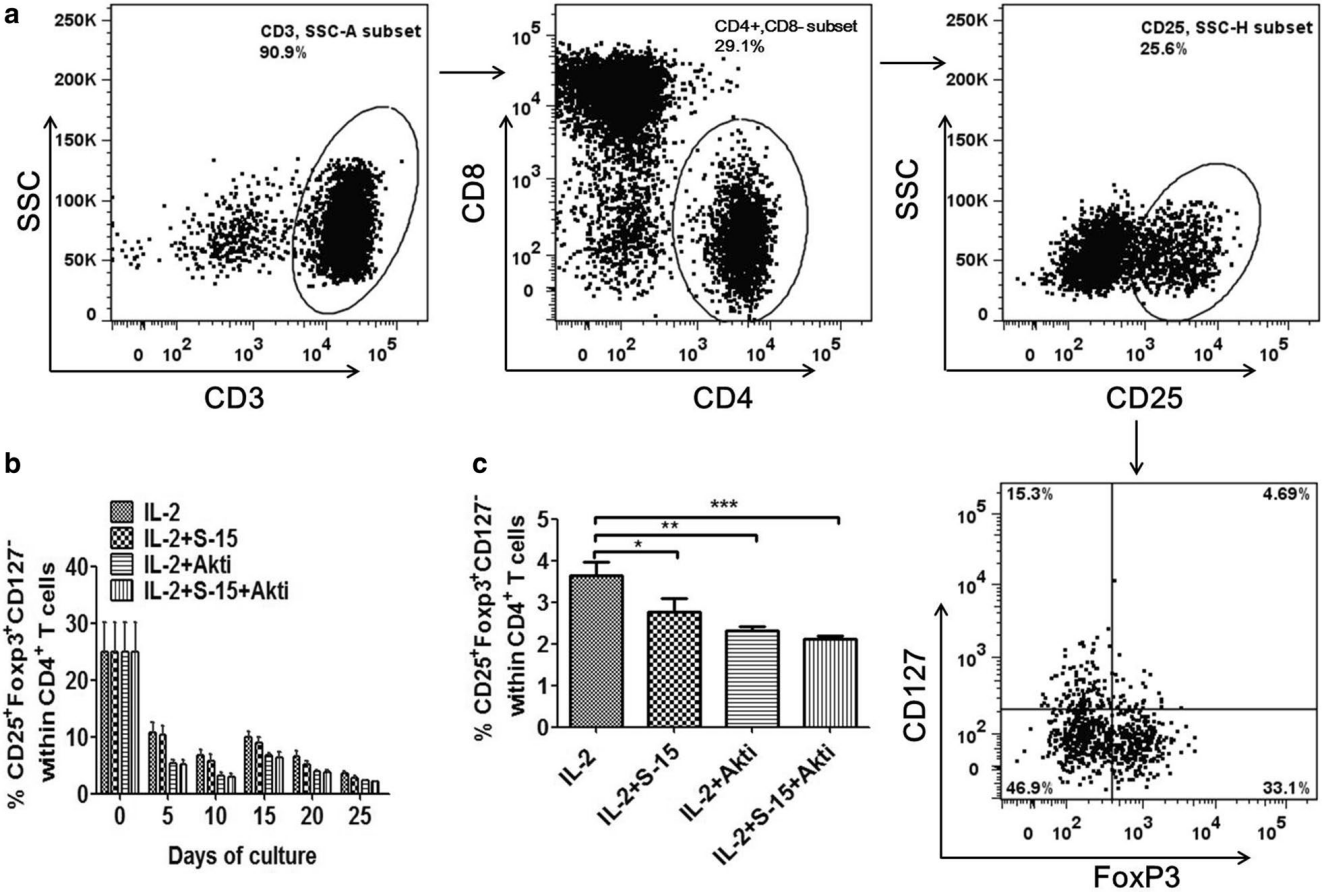
Expression of Tregs within CD4^+^ T cells in IL-2, IL-2/S-15, IL-2/Akti and IL-2/S-15/Akti-expanded Tils. **a** Tumor tissues were obtained from 6 HCC patients. Tissues were disaggregated as specified in “Materials and methods” section and stained with fluorochrome-labeled antibodies against CD3,CD4,CD8, CD25,CD127 and FoxP3. Acquired cells were first gated on CD3^+^ T cells, within CD3^+^ T cells the cells expressing CD4^+^CD8^−^ T cells were gated for next analysis, the cells express CD25^+^CD127^−^FoxP3^+^ within CD4^+^CD8^−^ T cells was determined as Tregs. Representative dot plots with percentages of CD25^+^CD127^−^FoxP3^+^ within CD4^+^ CD8^−^ T cells in different groups at day 25 are shown. **b**The dynamic percentages of Tregs within CD4^+^T cells during the 25-day initial culture period were shown. Data represent the mean ± SEM of six independent experiments. **c** Summary data about the percentages of Tregs within CD4^+^ CD8^−^ T cells at day 25 which is from panel **b** are presented. Statistical significance was analyzed by repeated measures ANOVA

Regulatory T cells (Tregs) play an important role in anticancer immunity. In the present study, CD4^+^CD25^+^CD127^−^ Foxp3^+^ were used to assess the Tregs among CD4^+^ T-cells from HCC patients. Prior to expansion, Tils had a higher Tregs up to 43.8% among CD4^+^ T-cells, it decreased prior to anti-CD3 antibody stimulation, but increased after anti-CD3 antibody stimulation, then it decreased gradually again during culture. The percentage of Tregs in IL-2 group was significantly higher than that in IL-2/S-15/Akti group (P < 0.001), IL-2/S-15 group (P < 0.05) and IL-2/Akti group (P < 0.01) at d25 (Fig. 4), but there was no difference between IL-2/Akti group and IL-2/S-15 group, as well as that between IL-2/Akti group and IL-2/S-15/Akti group.

### Combination of S-15 and Akt inhibition increases the IFN-γ producing tumor infiltrating CD8^+^ T cells

**Fig. 5:**
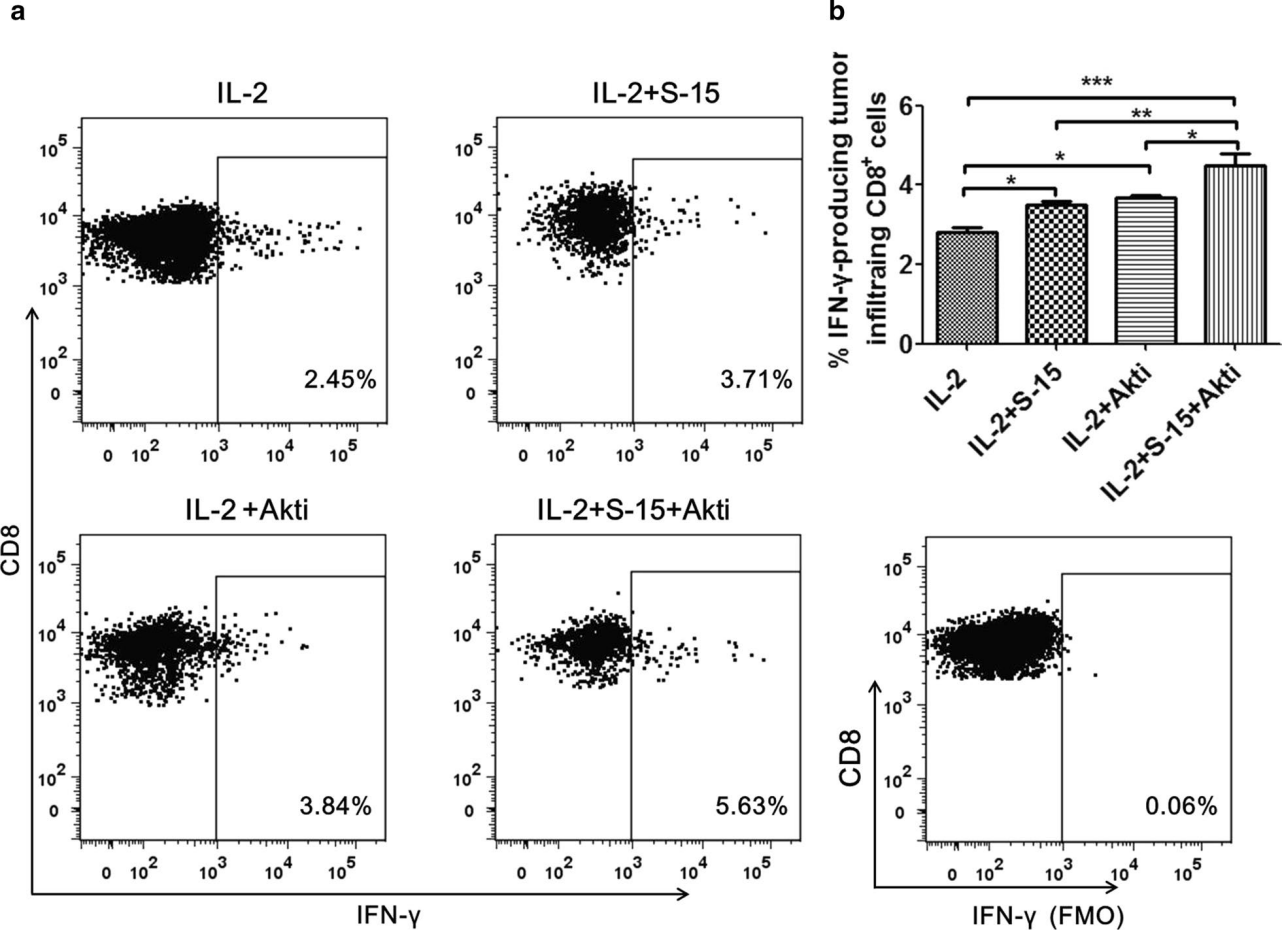
The percentage of IFN-γ-producing CD8^+^ T cells in IL-2, IL-2/S-15, IL-2/Akti and IL-2/S-15/Akti-expanded Tils. Representative dot plots from one HCC patient (**a**) and summary data for HCC patients (*n *= 6; **b**) showing the percentage of IFN-γ- producing CD8^+^ T cells after ex vivo co-culture with autologous tumor cells

To assess whether the Akti and S-15-expanded Tils retained tumor recognizing capabilities following expansion, the Tils obtained from different conditions were analyzed for IFN**-γ** production after co-culture with autologous tumor cells [11]. We observed the percentage of IFN**-γ** producing tumor infiltrating CD8^+^ T cells in IL-2/S-15/Akti group was significantly higher than that in IL-2 group (P < 0.001), IL-2/S-15 group (P < 0.01) and IL-2/Akti group (P < 0.05) at day 25 (Fig. 5). The percentage of IFN**-γ** producing tumor infiltrating CD8^+^ T cells in IL-2 group was significantly lower than that in IL-2/Akti group (P < 0.05) and IL-2/S-15 group (P < 0.05), but there was no significant difference between IL-2/Akti and IL-2/S-15 group.

## Discussion

Here we constructed S-15 successfully, and demonstrated that S-15 in combination of Akt inhibition promote the expression of CD45RA^−^CCR7^+^ and downregulates the expression of PD-1^+^Tim-3^+^ in human Tils from the patients with HCC, in addition S-15 in combination Akt inhibition decreases the Tregs in Tils and increases the IFN-γ producing tumor infiltrating CD8^+^ T cells without affecting their expansion.

In the present study, dynamic changes of Tils in the condition of IL-2, S-15 and Akti were observed. The expanded Tils were predominantly CD8^+^ T cells, this consistent with previously reported works by Dudley et al. [20] in which most melanoma-derived Tils cultures were predominantly CD8^+^ T cells, while there was a significant proportion of CD4^+^ T cells observed in Tils derived from epithelial ovarian cancer and glioma specimens [16, 21]. These results indicated that expanded Tils derived from different tumor tissues demonstrated highly heterogeneous ratios of CD4^+^:CD8^+^ T cells. The percentage of CD8^+^ cells among the CD3^+^ T cells on day 25 in IL-2/S-15 group was significant higher than that in IL-2 group which is consistent with our previous data that hyper-IL-15 provides an effective therapy against well-established liver cancers in mouse models by preferentially expanding tumor-specific CD8^+^ T cells [14, 15]. S-15 in combination with Akti had synergistic effect in upregulating the percentage of CD8^+^ T cells and decreasing the percentage of CD4^+^ cells among the CD3^+^ T cells.

There are considerable studies showed that adoptive cell therapy with central memory T cells (CD45RA^−^CCR7^+^) shown to exhibit stronger proliferative potential and mediate superior regression of tumor [2, 3]. Akt pathway and IL-15 both play a role in the regulation of T cell differentiation and memory formation [2–8, 22]. Our data shows that the percentages of Tcm either in CD8^+^ or CD4^+^ T cells both increased gradually during the culture in the condition of IL-2/S-15/Akti. The percentages of Tcm in IL-2 group were significantly lower than that in IL-2/Akti group. This consistently demonstrated that Akti and S-15-treated T cells both exhibited higher CD45RA^−^CCR7^+^ T cells. In addition, the percentages of Tcm in IL-2/S-15/Akti group were significantly higher than that in IL-2/S-15 group and IL-2/Akti group, it indicates that the combination of S-15 and Akti has a synergetic function to promoting central memory T cells without affecting their expansion.

The process of Tils activated and expanded in a long-term culture (about 25 days) in vitro is similar to a viral chronic infection and it may undergo exhaustion as characterized by co-expression of PD-1 and Tim-3. Consistent with our previous data that T cells within the tumor microenvironment are functionally suppressed [17, 19], Tils isolated from HCC have higher expression of PD-1^+^Tim-3^+^ T cells. PD-1 is a key immune-checkpoint receptor expressed by activated T cells which mediates the immunosuppression, there are also reports that expansion of tumor-infiltrating CD8^+^ T cells expressing PD-1 improves the efficacy of adoptive T-cell therapy [23]. During the in vitro culture, the expression of PD-1 decreased sharply at the early stage either on CD8^+^ or on CD4^+^ T cells because loss of the tumor microenvironment, then the expression PD-1 increased sharply because of stimulation by anti-CD3 antibody. However, the expression of PD-1 gradually decreases again after the short time increase. Tim-3 is generally considered to be a co-inhibitory receptor, while, our results showed that the expression of Tim-3 is upregulating sharply especially after anti-CD3 antibody stimulation and remained at a high level during long-term culture. This upregulation might be due to the activation of T cells. The dynamic changes of the expression of PD-1 and Tim-3 in the Tils were similar with that in the cytokine induced killers after stimulation by anti-CD3 antibody [24]. The dynamic changes of PD-1^+^Tim-3^+^ T cells were in according with the changes of PD-1^+^ T cells in Tils. The percentages of PD-1^+^Tim-3^+^ on CD8^+^ or CD4^+^ T cells decreased gradually after short-increased post anti-CD3 antibody stimulation. The mechanism of this phenomenon is still unclear. It may be associated with co-inhibitory receptors mediate distinct and synergistic effects on the activation of signaling pathways resulting in altered immune functions [25]. Further studies are required to address these questions.

The percentages of PD-1^+^Tim-3^+^ T cells among the CD8^+^ T cells on day 25 in IL-2/S-15/Akti group was significant lower than that in IL-2 group and IL-2/S-15 group. However, there was no difference between IL-2/S-15 group and IL-2/Akti group. Similar results were also found in the percentage PD-1^+^ or Tim-3^+^ T cells on CD8^+^ or CD4^+^ T cells [21]. Heon et al. reported that IL-15 induces strong but short-lived tumor-infiltrating CD8 T cell responses through the regulation of Tim-3 in breast cancer [10], in this study, there were no difference between IL-2 and IL-2/S-15 groups in regulation of Tim-3 on CD8^+^ or CD4^+^ T cells. In addition, Tim-3 expression correlates with senescent phenotype. Weng et al. reported that IL-15 enhances the antitumor effect of human antigen-specific CD8^+^ T cells by cellular senescence delay [26]. These results showed that S-15 in combination of Akti have synergistic effect in decreasing the exhausted cells within Tils, which are the properties required for efficient antitumor activity following adoptive transfer.

It is reported that IL-2 may lead to the expansion of suppressive Tregs during the culture of Tils, and this decreases the effect of the antitumor potency of the expanded Tils. Since activated T cells can also transiently express FoxP3, so CD25^+^CD127^−^ FoxP3^+^ was used to assess Tregs within CD4^+^ T-cells in our study. We found that the percentage of Tregs was decreased gradually at the early stage, but it showed a transiently upregulation after anti-CD3 antibody stimulation. This may be associated with the activation of the T cells and this upregulation has not been correlated with suppressive function, however, after day 15, the expression of Tregs gradually decreased, the underlying mechanism is still unclear. The percentage of Tregs within the CD4^+^ population from IL-2/S-15/Akti-expanded cultures was reduced compared to all other conditions. The percentage of Tregs within the CD4^+^ population from IL-2-expanded cultures were significant higher than that from IL-2/S-15 and IL-2/Akti-expanded cultures. This was consistent with findings from others describing a suppressive role for IL-15 and Akti on regulatory T cell expansion [10, 11]. On that note, the combination of S-15 and Akti has synergistic function in decreasing the Tregs.

The expanded Tils were analyzed for their cytotoxic potential by IFN**-γ** production after co-culture with autologous tumor cells. We also observed that S-15 or Akti can increase the percentage of IFN**-γ** producing tumor infiltrating CD8^+^ T cells. Interestingly, the IL-2/S-15/Akti-expanded Tils and demonstrated superior functional tumor recognition, as indicated by higher IFN-γ production levels than the Tils expanded by IL-2/S-15 and IL-2/Akti.

In summary, our findings demonstrate that IL-2/S-15/Akti combination is preferred for the expansion of T cells for adoptive T cell transfer, as they promote the expansion of CD45RA^−^CCR7^+^ tumor infiltrating lymphocytes with high cytotoxic potential and down-regulating PD-1^+^Tim-3^+^ cells and regulatory T cells.

## Conclusion

The data presented in this study suggest that S-15 in combination of Akt inhibitor promotes the expansion of CD45RA^−^CCR7^+^ tumor infiltrating lymphocytes with high cytotoxic potential and downregulating PD-1^+^Tim-3^+^ cells and regulatory T cells. We conclude that Tils expanded with IL-2/S-15/Akti combination is a viable source for the cellular therapy and a promising platform for the expansion of Tils use in clinical adoptive cell transfer trials.

## Supplementary information

**Additional file 1: Figure S1.** **(a)** Schematic representation of S-15(ptt3-hIL15Rαsushi-linker-hIL15-hIgGFc) constructs. For S-15 construction, the cDNA encoding human IL-15Ra-sushi domain (amino acids 25-89) and human IL-15 mature sequence were linked by a 20-amino acid linker and then fused with Fc.21. All the fragments were sub-cloned into the ptt3 plasmid. **(b)** Production and purification of human S-15. The proteins were prepared by transient transfection of 293T cells and purified by protein G columns. S-15 proteins were eluted from the column and analyzed by SDS-PAGE. Lane 1–4, bovine serum albumin(BSA) as control protein at 0.125 μg, 0.25 μg, 0.5 μg, 1 μg; lane 5, marker; lane 6, 15 μl the supernatant of 293T cells transfected with S-15; lane 7, 1 μl the concentrate supernatant of 293T cells transfected with S-15; lane 8, 5 μl purified S-15.

**Additional file 2: Figure S2.** Frequencies of CD45RA^−^CCR7^+^ among CD4^+^ T cells in IL-2, IL-2/S-15, IL-2/Akti and IL-2/S-15/Akti-expanded Tils. **(a)** The dynamic percentages of Tcm within CD4^+^ T cells during the 25-day initial culture period were shown. Data represent the mean ± SEM of six independent experiments. **(b)** Representative dot plots with percentages of CD45RA^−^CCR7^+^ among the CD4^+^ T cell population in different groups at day 25 are shown. **(c)** Summary data about the percentages of CD45RA^−^CCR7^+^ within the CD4^+^ T cell population at day 25 which is from panel a are presented. Statistical significance was analyzed by repeated measures ANOVA. *P < 0.05, **P < 0.01, ***P < 0.001.

**Additional file 3: Figure S3.** Expression of PD-1 and Tim-3 among CD4^+^ T cells in IL-2, IL-2/S-15, IL-2/Akti and IL-2/S-15/Akti-expanded Tils. **(a)** Representative dot plots with percentages of PD-1^+^Tim-3^+^ within CD4^+^T cells in different groups at day 25 are shown. **(b)** The dynamic percentages of PD-1^+^Tim-3^+^ within CD4^+^T cells during the 25-day initial culture period were shown. Data represent the mean ± SEM of six independent experiments. **(c)** Summary data about the percentages of PD-1^+^Tim-3^+^ within CD4^+^T cells at day 25 which is from panel b are presented. Statistical significance was analyzed by repeated measures ANOVA.

**Additional file 4: Figure S4.** Expression of PD-1 on T cells in IL-2, IL-2/S-15, IL-2/Akti and IL-2/S-15/Akti-expanded Tils. **(a)** The dynamic percentages of PD-1^+^ within CD4^+^T cells during the 25-day initial culture period were shown. Data represent the mean ± SEM of six independent experiments. **(b)** Summary data about the percentages of PD-1^+^ within CD4^+^T cells at day 25 which is from panel a are presented. **(c)** The dynamic percentages of PD-1^+^ within CD8^+^T cells during the 25-day initial culture period were shown. Data represent the mean ± SEM of six independent experiments. (d) Summary data about the percentages of PD-1^+^ within CD8^+^T cells at day 25 which is from panel c are presented. Statistical significance was analyzed by repeated measures ANOVA.

**Additional file 5: Figure S5.** Expression of Tim-3 on T cells in IL-2, IL-2/S-15, IL-2/Akti and IL-2/S-15/Akti-expanded Tils. **(a)** The dynamic percentages of Tim-3^+^ within CD4^+^ T cells during the 25-day initial culture period were shown. Data represent the mean ± SEM of six independent experiments. **(b)** Summary data about the percentages of Tim-3^+^ within CD4^+^ T cells at day 25 which is from panel a are presented. **(c)** The dynamic percentages of Tim-3^+^ within CD8^+^ T cells during the 25-day initial culture period were shown. Data represent the mean ± SEM of six independent experiments. (d) Summary data about the percentages of Tim-3^+^ within CD8^+^ T cells at day 25 which is from panel c presented. Statistical significance was analyzed by repeated measures ANOVA.

## Data Availability

Not applicable.

## Abbreviations

HCC: hepatocellular cancer
Tils: tumor-infiltrating lymphocytes
PD-1: programmed death 1
Tim-3: T cell immunoglobulin and mucin-domain-containing molecule 3
Tregs: regulatory T-cells
Tcm: central memory T cells
