# Supplementary figures and images for: S-15 in combination of Akt inhibitor promotes the expansion of CD45RA−CCR7+ tumor infiltrating lymphocytes with high cytotoxic potential and downregulating PD-1+Tim-3+ cells as well as regulatory T cells
Source: Cancer Cell Int. 2019 Dec 3;19:322. doi: 10.1186/s12935-019-1043-3 (PMC6889332; doi:10.1186/s12935-019-1043-3)

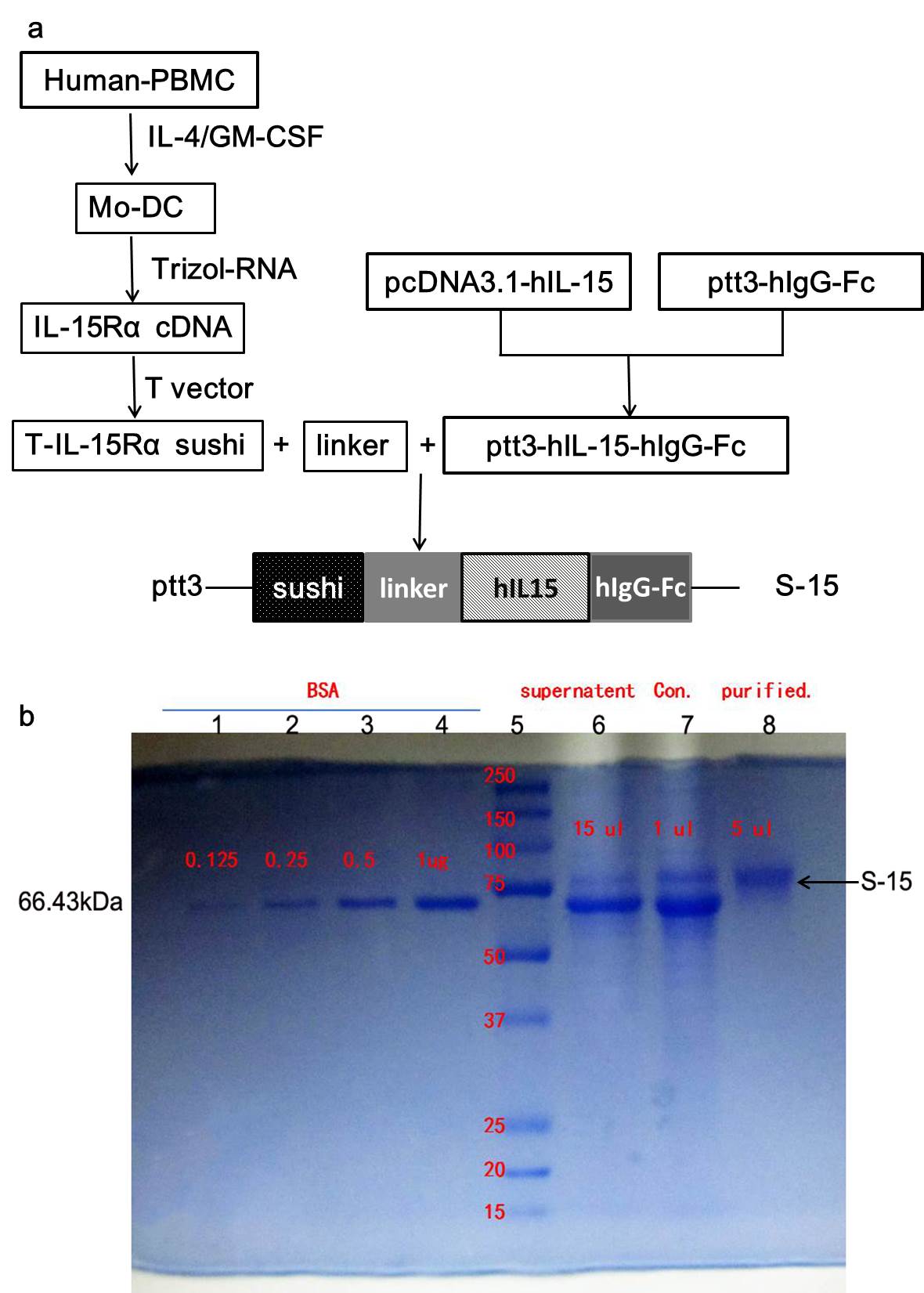

Supplement: Supplementary file 1 — Additional file 1: Figure S1. (a) Schematic representation of S-15(ptt3-hIL15Rαsushi-linker-hIL15-hIgGFc) constructs. For S-15 construction, the cDNA encoding human IL-15Ra-sushi domain (amino acids 25-89) and human IL-15 mature sequence were linked by a 20-amino acid linker and then fused with Fc.21. All the fragments were sub-cloned into the ptt3 plasmid. (b) Production and purification of human S-15. The proteins were prepared by transient transfection of 293T cells and purified by protein G columns. S-15 proteins were eluted from the column and analyzed by SDS-PAGE. Lane 1–4, bovine serum albumin(BSA) as control protein at 0.125 μg, 0.25 μg, 0.5 μg, 1 μg; lane 5, marker; lane 6, 15 μl the supernatant of 293T cells transfected with S-15; lane 7, 1 μl the concentrate supernatant of 293T cells transfected with S-15; lane 8, 5 μl purified S-15. [file 12935_2019_1043_MOESM1_ESM.jpg]

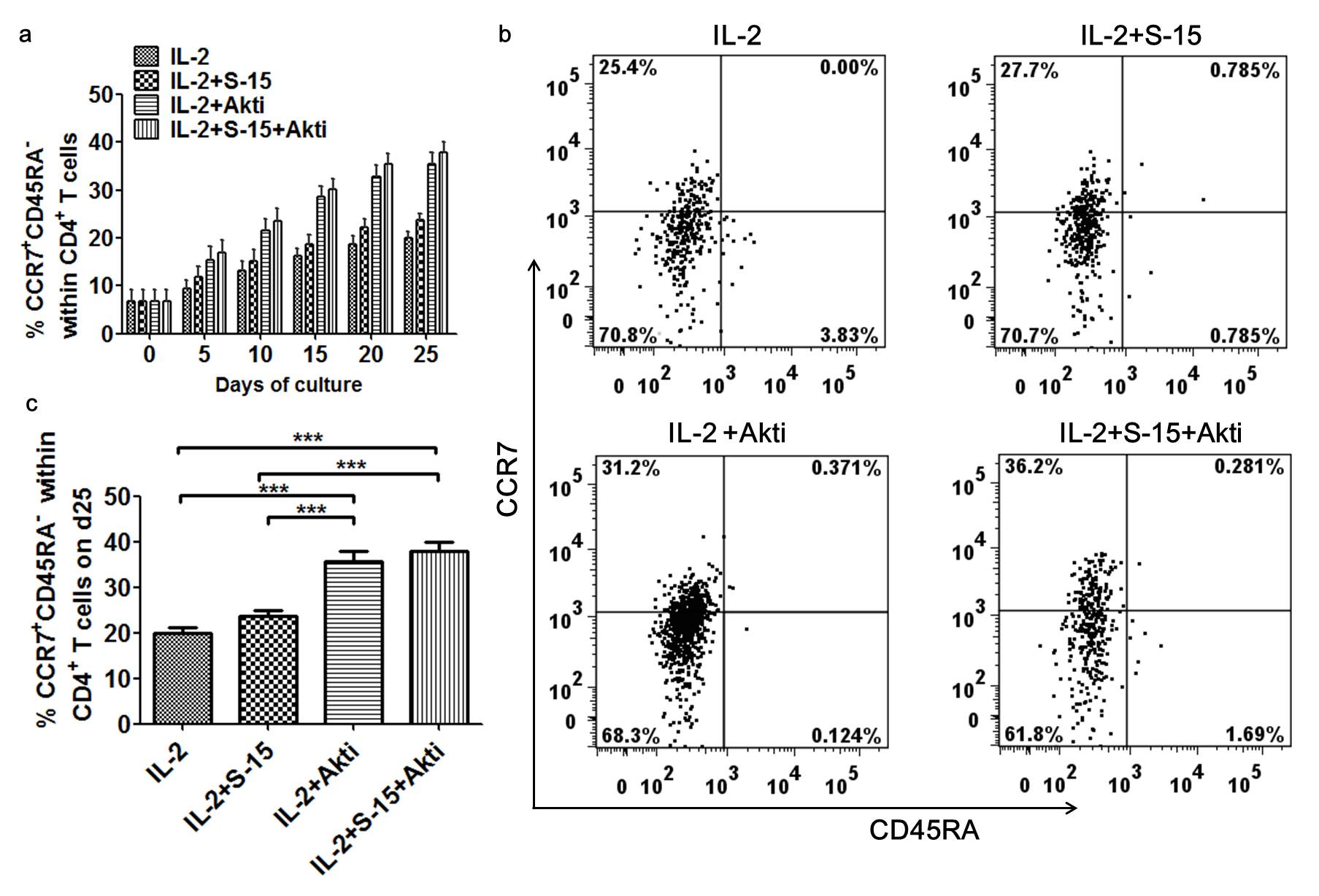

Supplement: Supplementary file 2 — Additional file 2: Figure S2. Frequencies of CD45RA−CCR7+ among CD4+ T cells in IL-2, IL-2/S-15, IL-2/Akti and IL-2/S-15/Akti-expanded Tils. (a) The dynamic percentages of Tcm within CD4+ T cells during the 25-day initial culture period were shown. Data represent the mean ± SEM of six independent experiments. (b) Representative dot plots with percentages of CD45RA−CCR7+ among the CD4+ T cell population in different groups at day 25 are shown. (c) Summary data about the percentages of CD45RA−CCR7+ within the CD4+ T cell population at day 25 which is from panel a are presented. Statistical significance was analyzed by repeated measures ANOVA. *P < 0.05, **P < 0.01, ***P < 0.001. [file 12935_2019_1043_MOESM2_ESM.jpg]

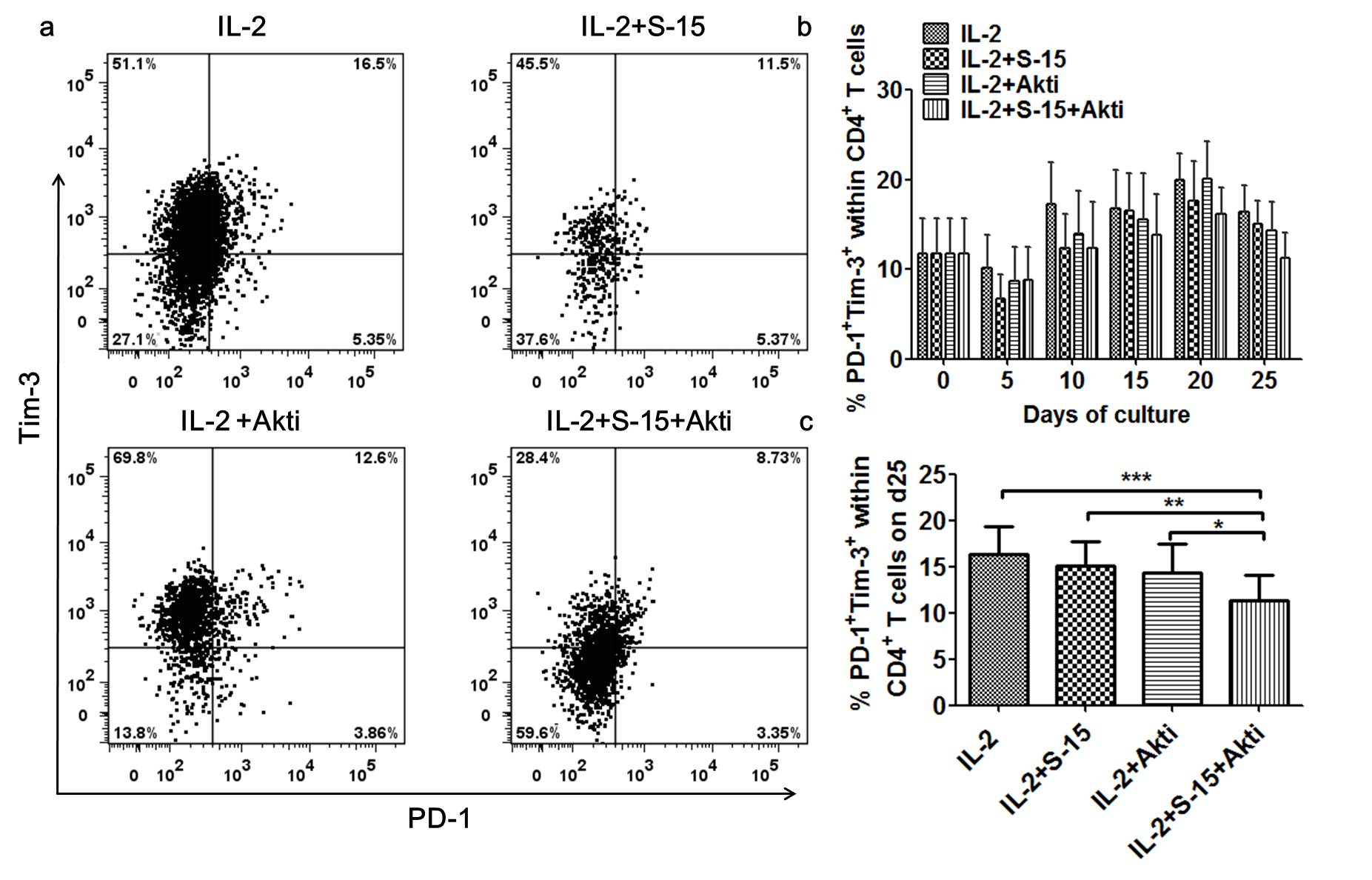

Supplement: Supplementary file 3 — Additional file 3: Figure S3. Expression of PD-1 and Tim-3 among CD4+ T cells in IL-2, IL-2/S-15, IL-2/Akti and IL-2/S-15/Akti-expanded Tils. (a) Representative dot plots with percentages of PD-1+Tim-3+ within CD4+T cells in different groups at day 25 are shown. (b) The dynamic percentages of PD-1+Tim-3+ within CD4+T cells during the 25-day initial culture period were shown. Data represent the mean ± SEM of six independent experiments. (c) Summary data about the percentages of PD-1+Tim-3+ within CD4+T cells at day 25 which is from panel b are presented. Statistical significance was analyzed by repeated measures ANOVA. [file 12935_2019_1043_MOESM3_ESM.jpg]

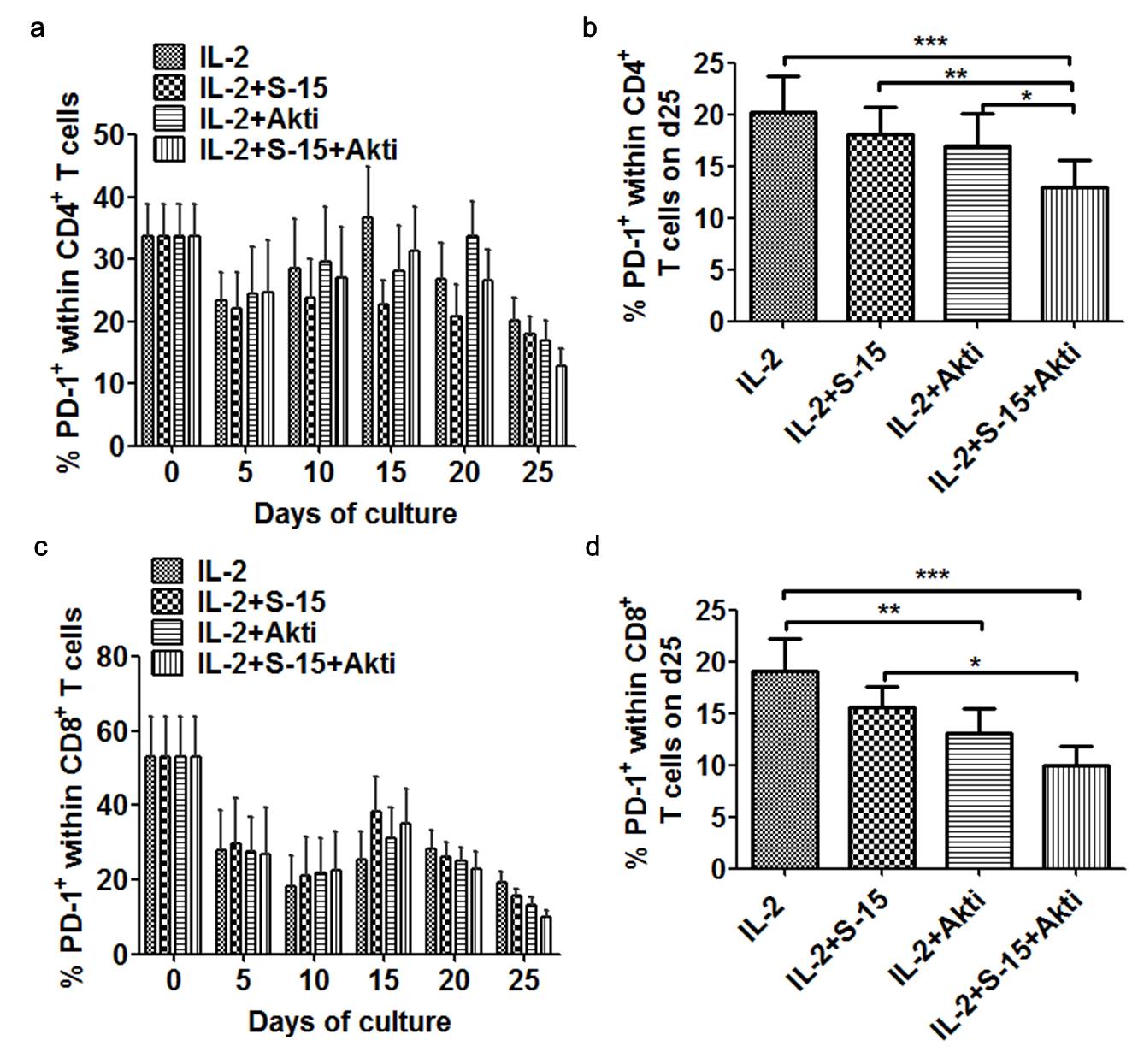

Supplement: Supplementary file 4 — Additional file 4: Figure S4. Expression of PD-1 on T cells in IL-2, IL-2/S-15, IL-2/Akti and IL-2/S-15/Akti-expanded Tils. (a) The dynamic percentages of PD-1+ within CD4+T cells during the 25-day initial culture period were shown. Data represent the mean ± SEM of six independent experiments. (b) Summary data about the percentages of PD-1+ within CD4+T cells at day 25 which is from panel a are presented. (c) The dynamic percentages of PD-1+ within CD8+T cells during the 25-day initial culture period were shown. Data represent the mean ± SEM of six independent experiments. (d) Summary data about the percentages of PD-1+ within CD8+T cells at day 25 which is from panel c are presented. Statistical significance was analyzed by repeated measures ANOVA. [file 12935_2019_1043_MOESM4_ESM.jpg]

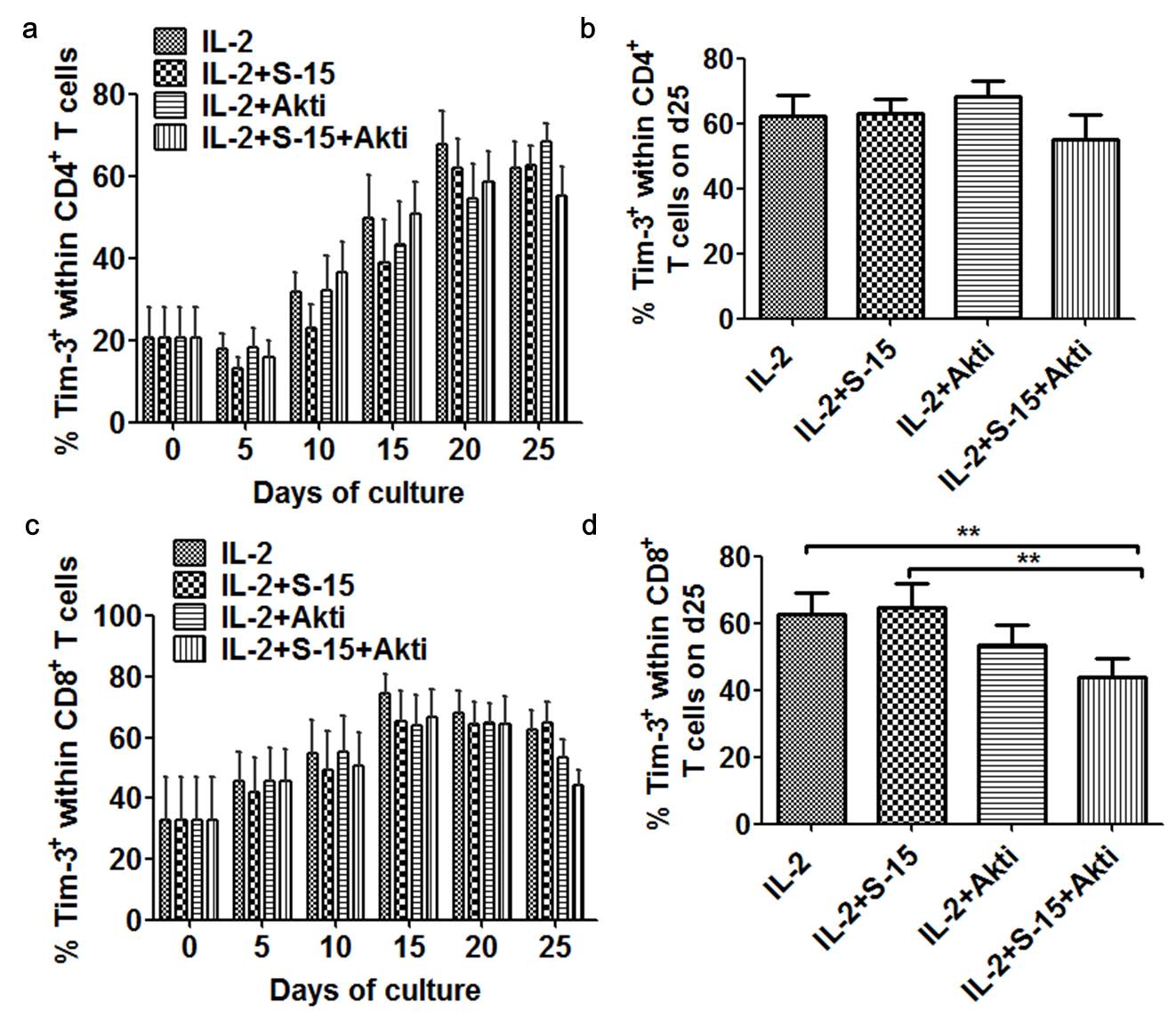

Supplement: Supplementary file 5 — Additional file 5: Figure S5. Expression of Tim-3 on T cells in IL-2, IL-2/S-15, IL-2/Akti and IL-2/S-15/Akti-expanded Tils. (a) The dynamic percentages of Tim-3+ within CD4+ T cells during the 25-day initial culture period were shown. Data represent the mean ± SEM of six independent experiments. (b) Summary data about the percentages of Tim-3+ within CD4+ T cells at day 25 which is from panel a are presented. (c) The dynamic percentages of Tim-3+ within CD8+ T cells during the 25-day initial culture period were shown. Data represent the mean ± SEM of six independent experiments. (d) Summary data about the percentages of Tim-3+ within CD8+ T cells at day 25 which is from panel c presented. Statistical significance was analyzed by repeated measures ANOVA. [file 12935_2019_1043_MOESM5_ESM.jpg]
